# Integrated proteogenomic analysis for inherited bone marrow failure syndrome

**DOI:** 10.1038/s41375-024-02263-1

**Published:** 2024-05-13

**Authors:** Manabu Wakamatsu, Hideki Muramatsu, Hironori Sato, Masaki Ishikawa, Ryo Konno, Daisuke Nakajima, Motoharu Hamada, Yusuke Okuno, Yusuke Kawashima, Asahito Hama, Masafumi Ito, Hideto Iwafuchi, Yoshiyuki Takahashi, Osamu Ohara

**Affiliations:** 1 Department of Pediatrics, Nagoya University Graduate School of Medicine, Showa-ku, Nagoya 466-8560 Japan; 2 Department of Applied Genomics, Kazusa DNA Research Institute, Kisarazu, Chiba 292-0818 Japan; 3 Department of Pediatrics, Chiba University Graduate School of Medicine, Chuo-ku, Chiba 260-8670 Japan; 4 Department of Virology, Nagoya City University Graduate School of Medical Sciences, Mizuho-ku, Nagoya 464-0083 Japan; 5 Department of Hematology and Oncology, Children’s Medical Center, Japanese Red Cross Aichi Medical Center Nagoya First Hospital, Nakamura-ku, Nagoya 453-8511 Japan; 6 Department of Pathology, Japanese Red Cross Aichi Medical Center Nagoya First Hospital, Nakamura-ku, Nagoya 453-8511 Japan; 7 Department of Pathology, Shizuoka Children’s Hospital, Aoi-ku, Shizuoka 420-095 Japan

**Keywords:** Myelodysplastic syndrome, Acute myeloid leukaemia

## Abstract

Recent advances in in-depth data-independent acquisition proteomic analysis have enabled comprehensive quantitative analysis of >10,000 proteins. Herein, an integrated proteogenomic analysis for inherited bone marrow failure syndrome (IBMFS) was performed to reveal their biological features and to develop a proteomic-based diagnostic assay in the discovery cohort; dyskeratosis congenita (*n* = 12), Fanconi anemia (*n* = 11), Diamond–Blackfan anemia (DBA, *n* = 9), Shwachman–Diamond syndrome (SDS, *n* = 6), ADH5/ALDH2 deficiency (*n* = 4), and other IBMFS (*n* = 18). Unsupervised proteomic clustering identified eight independent clusters (C1–C8), with the ribosomal pathway specifically downregulated in C1 and C2, enriched for DBA and SDS, respectively. Six patients with SDS had significantly decreased SBDS protein expression, with two of these not diagnosed by DNA sequencing alone. Four patients with ADH5/ALDH2 deficiency showed significantly reduced ADH5 protein expression. To perform a large-scale rapid IBMFS screening, targeted proteomic analysis was performed on 417 samples from patients with IBMFS-related hematological disorders (*n* = 390) and healthy controls (*n* = 27). SBDS and ADH5 protein expressions were significantly reduced in SDS and ADH5/ALDH2 deficiency, respectively. The clinical application of this first integrated proteogenomic analysis would be useful for the diagnosis and screening of IBMFS, where appropriate clinical screening tests are lacking.

## Introduction

Inherited bone marrow failure syndrome (IBMFS) is a heterogeneous group of disorders characterized by cytopenia in at least one hematopoietic cell lineage, which may progress to pancytopenia and be considered as a predisposition to developing hematological malignancy or solid tumor [1, 2]. Its genetic etiology consists of germline variants in >30 distinct types of disorders, including Shwachman–Diamond syndrome (SDS), Fanconi anemia (FA), dyskeratosis congenita (DC), Diamond–Blackfan anemia (DBA), and recently identified alcohol dehydrogenase 5/aldehyde dehydrogenase 2 (ADH5/ALDH2) deficiency [3–6]. Next-generation sequencing (NGS) analysis has greatly enhanced the elucidation of underlying disease mechanisms in IBMFS, consequently improving the clinical management and genetic counseling for patients with IBMFS [7–9]. However, the causative genes still could not be identified in >50% of patients with IBMFS, requiring the establishment of another diagnostic tool to complement with genetic analysis.

SDS is characterized by pancreatic exocrine abnormalities, cytopenia, and skeletal abnormalities, and 15–30% of SDS cases progress to myelodysplastic syndrome (MDS) and acute myeloid leukemia (AML) [10]. Approximately 90% of patients with SDS are caused by biallelic variants in the *SBDS* gene involved in ribosome production [11]. *SBDS* variants are sometimes overlooked in short-read DNA sequencing (DNA-seq) because of an *SBDSP1* pseudogene with 97% homology [12], complicating the identification of pathogenic *SBDS* variants or the estimation of these allele fractions [13]. In patients clinically suspected with SDS, the *SBDS* gene should be assessed with Sanger sequencing using long polymerase chain reaction or long-read NGS analysis and/or the SBDS protein expression by Western blotting.

Recent studies have described ADH5/ALDH2 deficiency as ADD syndrome or AMeD syndrome, a digenic disorder belonging to IBMFS. This condition is caused by a defect in the endogenous formaldehyde-directed catabolic system caused by digenic pathogenic mutants of the *ADH5* and *ALDH2* genes [4, 5]. It is characterized by short stature, mental retardation, pancytopenia, and progression to MDS. These clinical manifestations are similar to those in FA based on several aspects. Unlike in FA, chromosome breakage analysis with the addition of mitomycin C is normal in ADH5/ALDH2 deficiency, and the DNA repair capacity is not impaired [4, 5]. The *ALDH2* gene variant associated with this clinical condition is a common single nucleotide polymorphism (SNP) (c.1510G>A, p.E504K [rs671]) present in 25.5% of East Asians [14]. Thus, this syndrome should be accurately identified particularly in patients suspected of IBMFS in Asian countries.

A recent remarkable progress in in-depth data-independent acquisition (DIA) proteomic analysis facilitates comprehensive quantitative analysis of >10,000 proteins, including extremely low-abundant ones, such as kinases and transcription factors [15, 16]. Several preceding proteomic analyses in oncology, such as brain tumors or clear cell renal cell carcinomas, integrated with other omics data have provided underlying molecular mechanisms and a biological perspective of subgroups beyond traditional histological boundaries [17, 18]. To the best of our knowledge, a large-scale proteomic analysis of various IBMFS and their integration with genomic and transcriptomic analyses has not been performed yet. In this study, we performed a multi-omics analysis for patients enrolled in the IBMFS registry in Japan to evaluate the potential significance of proteomic analysis in diagnosing and pathophysiologically evaluating IBMFS.

## Materials and methods

### Patients and samples

In this study, 77 patients with IBMFS who underwent target-captured DNA-seq analysis at Nagoya University from December 2013 to March 2020 were enrolled. The enrolled 77 patients with IBMFS were diagnosed based on the published diagnostic criteria for IBMFS, MDS, and acquired aplastic anemia (AA) [10, 19]. “IBMFS, not otherwise specified (NOS)” was defined as follows: suspicion of IBMFS based on clinical features (physical [growth] or organ abnormalities [skin, nails, hair, bones, heart, lung, liver, and genitourinary]), a family history of blood disorders, early onset (<2 years), short telomere length (<2.0 SD), and hypersensitivity to chromosome breakage analysis, but with the absence of IBMFS-related pathogenic variants. All 77 patients with IBMFS underwent in-depth non-targeted proteomic analysis using their peripheral blood mononuclear cells (PBMC, *n* = 60; the discovery cohort) (Table 1) and/or bone marrow mononuclear cells (BMNC, *n* = 18) (Supplementary Table 1). Concurrently, the same analyses were performed using PBMC from 14 healthy individuals as controls.

**Table 1:** Patient characteristics in the discovery cohort.

Proteogenomic diagnosis	SDS^a^ (*n* = 6)	BMFS with monoallelic *SBDS* variant (*n* = 6)	ADH5/ALDH2 deficiency (*n* = 4)	DC (*n* = 12)	FA (*n* = 11)	DBA (*n* = 9)	IBMFS, NOS (*n* = 12)
Age at diagnosis (range), (years)	2.4 (0–23)	1.1 (0–3)	5.0 (3–8)	14.2 (1–19)	1.7 (0–7)	1.0 (0–13)	1.3 (0–19)
Gender, *n* (%)							
Male/Female	3 (50)/3 (50)	2 (33)/4 (67)	1 (25)/3 (75)	6 (50)/6 (50)	8 (73)/3 (27)	3 (33)/6 (67)	4 (33)/8 (67)
WBC, median (range), (×10^9^/L)	4.0 (2.6–8.4)	7.2 (1.9–12.0)	3.8 (1.9–9.8)	3.3 (1.7–8.3)	5.1 (1.7–9.8)	4.8 (2.1–10.9)	3.9 (2.4–12.8)
Hb, median (range), (g/dL)	8.5 (5.1–12.5)	11.4 (8.0–12.6)	10.2 (7.7–10.7)	9.6 (3.1–14.7)	10.1 (8.4–13.1)	7.2 (4.7–13.2)	5.8 (2.3–14.3)
ANC, median (range), (×10^9^/L)	0.3 (0.09–1.4)	0.9 (0.0–5.3)	0.5 (0.1–3.9)	0.9 (0.6–2.2)	1.2 (0.3–2.8)	1.8 (0.3–2.9)	1.5 (0.05–5.3)
Plt, median (range), (×10^9^/L)	124 (29–155)	177 (37–570)	58 (24–77)	30 (3–238)	45 (18–130)	409 (16–590)	52 (8–421)
Ret, median (range), (‰)	10.3 (3.8–16.9)	15.1 (3.0–19.0)	14.4 (5.0–24.0)	23.5 (8.0–32.1)	19.0 (2.5–39.1)	3.0 (1.0–28.0)	8.3 (0.5–57.0)
Relative telomere length, (SD)							
Median (range)	−0.14 (−1.99 to +1.71)	−1.50 (−1.60 to −0.93)	−0.01 (−0.87 to +1.24)	−2.57 (−5.70 to +0.83)	+0.48 (−0.26 to +2.05)	−0.82 (−1.37 to +1.21)	−2.00 (−3.20 to +2.39)
Pathological diagnosis, (‰)							
MDS	1 (17)	0 (0)	3 (75)	4 (33)	5 (46)	0 (0)	4 (33)
Non-MDS/Unknown	5 (83)	6 (100)	1 (25)	8 (67)	6 (54)	9 (100)	8 (67)
Monosomy 7, *n* (%)							
Positive	1 (16)	0 (0)	2 (50)	0 (0)	2 (18)	0 (0)	2 (16)
Negative/Unknown	5 (84)	6 (100)	2 (50)	12 (100)	9 (82)	9 (100)	10 (84)
Trisomy 8, *n* (%)							
Positive	1 (16)	0 (0)	1 (25)	0 (0)	1 (9)	0 (0)	1 (8)
Negative/Unknown	5 (84)	6 (100)	3 (75)	12 (100)	10 (91)	9 (100)	11 (92)
Chromosomal abnormalities, *n* (%)							
Yes	2 (33)	0 (0)	3 (75)	1 (8)	0 (0)	0 (0)	1 (8)
No/Unknown	4 (67)	6 (100)	1 (0)	11 (92)	11 (100)	9 (100)	11 (92)
Chromosome breakage analysis, *n* (%)							
Abnormal	1 (16)	0 (0)	1 (25)	1 (8)	10 (91)	0 (0)	0 (0)
Normal/Unknown	5 (84)	6 (100)	3 (75)	11 (92)	1 (9)	9 (100)	12 (100)
Clinical diagnosis, *n* (%)							
SDS	2 (33)	2 (33)	0 (0)	0 (0)	0 (0)	0 (0)	0 (0)
DC	0 (0)	0 (0)	0 (0)	4 (34)	0 (0)	1 (11)	1 (8)
FA	0 (0)	0 (0)	1 (25)	2 (17)	7 (64)	0 (0)	0 (0)
DBA	0 (0)	0 (0)	0 (0)	0 (0)	0 (0)	8 (89)	2 (17)
AA	0 (0)	0 (0)	0 (0)	5 (42)	0 (0)	0 (0)	3 (25)
MDS	2 (33)	0 (0)	3 (75)	0 (0)	1 (9)	0 (0)	4 (34)
IBMFS	2 (33)	4 (67)	0 (0)	1 (9)	3 (27)	0 (0)	2 (17)

*AA* aplastic anemia, *AML* acute myeloid leukemia, *ANC* absolute neutrophil count, *DBA* Diamond–Blackfan anemia, *DC* dyskeratosis congenita, *FA* Fanconi anemia, *IBMFS* inherited bone marrow failure syndrome, *MDS* myelodysplastic syndrome, *NOS* not otherwise specified, *Plt* platelet count, *Ret* reticulocyte count, *SD* Standard deviation, *SDS* Shwachman–Diamond syndrome, *WBC* white blood cell.

^a^Includes two patients who only presented with the monoallelic *SBDS* variant on DNA sequencing and a decreased expression of SBDS proteins based on the proteomic analysis. These patients were finally diagnosed with SDS. In these two cases, biallelic *SBDS* variants were identified after a detailed re-evaluation of the RNA sequencing read alignment.

Subsequently, to establish a simplified rapid screening testing for IBMFS, the targeted protein expression levels (SBDS, ADH5, and WAS) in 417 samples (PBMC, *n* = 401; BMNC, *n* = 16) from patients with IBMFS-related hematological disorders (*n* = 390) and healthy controls (*n* = 27) was measured (Supplementary Table 2). Written informed consent was obtained from patients or their guardians, and the study was approved by the ethics committee of the Nagoya University Graduate School of Medicine.

### In-depth non-targeted proteomic analysis

The frozen PBMC or BMNC suspensions of patients were rapidly thawed in a 37 °C water bath and carefully washed twice with phosphate-buffered saline, and then, the RNAs and proteins were simultaneously isolated from the same biological sample using the TRIzol reagent (Thermo Fisher Scientific, Waltham, MA) [20]. Protein fractionation was dissolved in 100 mM Tris-HCl (pH 8.5) containing 2% SDS using a water bath-type sonicator (Bioruptor II, CosmoBio, Tokyo Japan). The pretreatment for shotgun proteome analysis was performed as previously reported [15]. The digested peptides were directly injected onto a 75 μm × 12 cm nanoLC nano-capillary column (Nikkyo Technos Co., Ltd., Tokyo, Japan) at 40 °C and then separated with an 80 min gradient at 150 nL/min using an UltiMate 3000 RSLCnano LC system (Thermo Fisher Scientific). Peptides eluting from the column were analyzed on a Q Exactive HF-X (Thermo Fisher Scientific) for overlapping window data-independent acquisition mass spectrometry (DIA-MS) [16, 21]. For DIA-MS, MS1 spectra were collected from 495 to 785 m/z at 30,000 resolutions to set an automatic gain control (AGC) target of 3.0 × 10^6^. The MS2 spectra were collected from >200 m/z at 30,000 resolutions to set an AGC target of 3.0 × 10^6^, a maximum injection time of “auto,” and stepped normalized collision energies of 22%, 26%, and 28%. An isolation width for MS2 was set to 4 m/z, and overlapping window patterns in 500–780 m/z were used as window placements optimized by Skyline ver. 4.1 [22].

### Targeted proteomic analysis

The targeted proteomic analysis was performed to generate a small target panel, comprising SBDS, ADH5, WASP, and GAPDH proteins. Targeted proteomic analysis was performed using the SureQuant method [23]. This method is based on the data-dependent acquisition MS (DDA-MS) and stable isotope-labeled (SIL) peptides that trigger the fragmentation of the corresponding endogenous peptides. In brief, SIL peptides of the targets were spiked in the samples; 0.4 pmol of SBDS SIL, 1.0 pmol of two ADH5 SILs, 0.4 pmol of two WASP SILs, and 0.4 pmol of two GAPDH SILs (Cosmo Bio Co., Ltd., Tokyo, Japan) were used in this study (Supplementary Table 3). Subsequently, the samples were measured by DDA-MS with SIL offset triggered fragmentation. For DDA-MS, MS1 spectra were collected, ranging from 450 to 1200 m/z at 60,000 resolutions to set an AGC target of 3.0 × 10^6^. The MS2 spectra were collected in the range of 200–1800 m/z at 60,000 resolutions to set an AGC target of 1 × 10^6^ collision induced the dissociation with a normalized collision energy of 28%. In the present study, the SIL offset triggered fragmentation and intensity threshold were set at least four ions (Supplementary Table 3) and 1.0 × 10^5^, respectively. All samples were analyzed using an Orbitrap Exploris 480 mass spectrometer (Thermo Fisher Scientific) equipped with an EVOSEP ONE system (EVOSEP, Odense, Danmark). As detailed methods of the EVOSEP One acquisition are described elsewhere [24], digested peptides with SILs were loaded onto the Evotip (EVOSEP) based on the manufacturer’s protocol and washed using 20 μL of 0.1% formic acid. MS data were analyzed using Skyline ver. 4.1 [22]. Each protein expression level was calculated based on GAPDH protein expression levels.

### Data processing for in-depth non-targeted proteomic analysis

Mass spectrometry results were retrieved using Scaffold DIA (version 2.2.1, Proteome Software, Inc., Portland, OR, USA) against the human spectral library, generated from the human protein sequence database (ID UP000005640, reviewed, canonical; https://www.uniprot.org/proteomes/UP000005640). The protein identification threshold was set at a false discovery rate (FDR) of <1% for peptides and proteins. The Scaffold DIA search parameters included trypsin as an experimental data search enzyme, 1 as the maximum missed cleavage sites, 8 ppm as precursor mass tolerance, 8 ppm as fragment mass tolerance, and cysteine carbamidomethylation as static modification. The Encyclope DIA algorithm was used for peptide quantification in Scaffold DIA to normalize protein quantification values between samples and to calculate the total quantification value.

Non-targeted proteomic data used protein expression levels where ≥3 peptides could be identified and were present in at least 70% of samples in each IBMFS group. Missing protein values were replaced by random numbers based on a normal distribution. Identified proteins were characterized based on the Human Protein Atlas and the Human Body Fluid Proteome [25]. Between-group comparisons were set up by filtering the data and then performing differential protein expression analysis using the R package DESeq2 (version 4.2) [26]. The Kyoto Encyclopedia of Genes and Genomes (KEGG) and Gene Ontology (GO) databases were used to classify and group candidate proteins. The KEGG pathways and GO with corrected *P* of <0.05 were regarded as significant. Pathway scores were computed based on single-sample gene set enrichment analysis (ssGSEA) values using the R package gene set variation analysis (version 1.47.0) [27].

## Results

### Clinical and genetic diagnoses of the discovery cohort

**Fig. 1:**
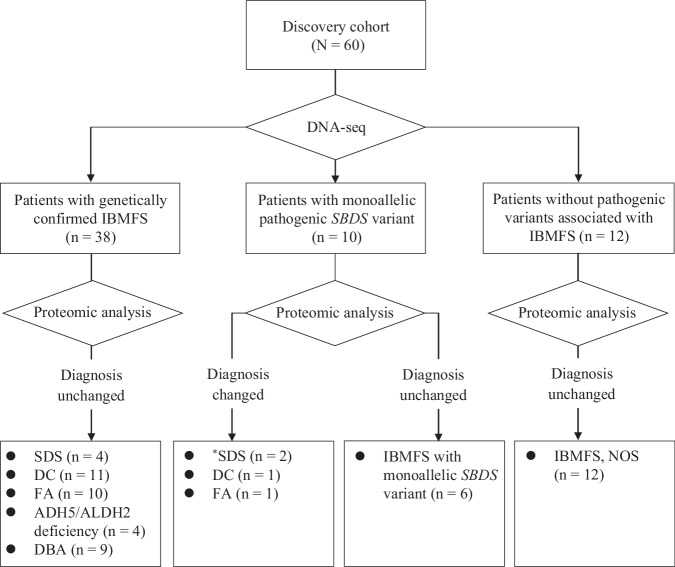
Diagnostic flowchart based on the proteogenomic analysis. Of the 10 patients with monoallelic *SBDS* variant detected by DNA sequencing (DNA-seq), four had diagnostic changes after a non-targeted proteomic analysis. Two patients with a significantly reduced SBDS protein expression were finally diagnosed with SDS, one with DC, and one with FA. ^*^The two patients with SDS only carried the monoallelic *SBDS* variant detected by DNA-seq. However, proteomic analysis revealed a reduced SBDS protein expression, and subsequent comprehensive RNA-seq read alignment evaluation confirmed the presence of biallelic *SBDS* variants. ADH5 alcohol dehydrogenase 5, ALDH2 aldehyde dehydrogenase 2, DBA Diamond–Blackfan anemia, DC dyskeratosis congenita, FA Fanconi anemia, IBMFS inherited bone marrow failure syndrome, NOS not otherwise specified, SDS Shwachman–Diamond syndrome.

**Fig. 2:**
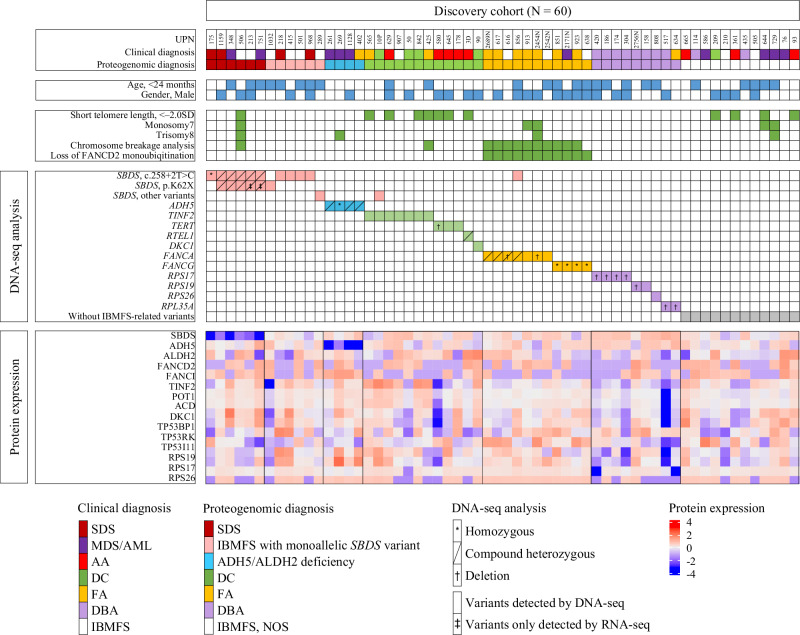
Proteomic and genomic profiling of the discovery cohort. Patient characteristics of the IBMFS discovery cohort. An omics analysis of target-captured or whole-exome DNA, in-depth proteomic, and transcriptomic analyses was conducted in patients with IBMFS (*N* = 60) as the discovery cohort. Proteomic analysis identified six patients with significantly reduced SBDS protein expression; however, two of them were undiagnosed with DNA-seq analysis only. The ADH5 protein expression was significantly reduced in ADH5/ALDH2 deficiency. ADH5 alcohol dehydrogenase 5, ALDH2 aldehyde dehydrogenase 2, AML acute myeloid leukemia, ANC absolute neutrophil counts, DBA Diamond–Blackfan anemia, DC dyskeratosis congenita, FA Fanconi anemia, Hb hemoglobin, IBMFS inherited bone marrow failure syndrome, MDS myelodysplastic syndrome, NOS not otherwise specified, PBMC peripheral blood mononuclear cells, Plt platelet count, Ret reticulocyte count, SD standard deviation, SDS Shwachman–Diamond syndrome, WBC white blood cell.

The clinical characteristics of the discovery cohort (*n* = 60) are shown in Table 1. In this cohort, 27 patients were men, with a median age at disease onset of 3 (range: 0–23) years. The median (range) white blood cell count, hemoglobin level, neutrophil count, platelet count, and reticulocyte count were 4.2 × 10^9^/L (1.7–12.8 × 10^9^/L), 9.6 g/dL (3.1–14.7 g/dL), 1.2 × 10^9^/L (0.0–5.3 × 10^9^/L), 63 × 10^9^/L (3–590 × 10^9^/L), and 13.0‰ (1.0–57.0‰), respectively. In the discovery cohort, target-captured DNA-seq analysis identified 38 patients diagnosed with a genetically confirmed IBMFS, 10 with a monoallelic pathogenic *SBDS* variant, and 12 without a pathogenic variant associated with IBMFS (Fig. 1). The definitive diagnoses of 38 patients with IBMFS included SDS (*n* = 4), ADH5/ALDH2 deficiency (*n* = 4), DC (*n* = 11), FA (*n* = 10), and DBA (*n* = 9). The identified pathogenic variants in the discovery cohort are shown in Supplementary Table 4. Among the four patients with a confirmed SDS diagnosis by DNA-seq, three had compound heterozygous *SBDS* variants (c.258+2T>C and c.183_184delTAinsCT, p.K62X) and one had a homozygous *SBDS* splice site variant (c.258+2T>C) (Fig. 2). Of the 10 patients with monoallelic pathogenic *SBDS* variant, 8, 1, and one patient had splice site variants (c.258+2T>C), a nonsense variant (p.K62X), and a missense variant (c.97A>G, p.K33E), respectively. In total, 2 of 10 patients had a reduced SBDS protein expression based on the proteomic analysis, and RNA-seq read alignment identified compound heterozygous variants. Hence, these two patients were finally diagnosed with SDS. Supplementary Table 5 presents the clinical characteristics of patients with monoallelic or biallelic *SBDS* variants. Of the 12 patients with DC detected with pathogenic variants in the telomere-related genes, seven had short telomere lengths. Further, three had normal telomere lengths, and the remaining two had missing data. Eleven patients with FA harbored the monoallelic or biallelic pathogenic *FANCA* (*n* = 7) or *FANCG* (*n* = 4) variants. Among them, 10 tested positive for chromosome breakage analysis and the remaining one patient had missing data. Of nine patients with DBA, *RPS17*, *RPL35A*, and *RPS19* deletions were identified in 4, 2, and 1 patients, respectively, and the remaining two patients had pathogenic variants of *RPS19* and *RPS26*. Patients with DBA had significantly lower reticulocyte counts than those without DBA (*P* = 0.04).

The extension cohort (*n* = 330) included patients with MDS/AML (*n* = 113), AA (*n* = 75), FA (*n* = 12), DBA (*n* = 11), DC (*n* = 7), SDS (*n* = 1), Wiskott–Aldrich syndrome (WAS, *n* = 6), and IBMFS, NOS (*n* = 105) (Supplementary Table 2). In this cohort, 51 (15.4%) patients presented with karyotypic abnormalities, and 22 (6.6%) had a family history. Moreover, 11 (3.3%) patients tested positive for chromosome breakage analysis. All patients in the extension cohort underwent targeted proteomic analysis with the IBMFS-related small panel.

### Integration of in-depth non-targeted proteomic and transcriptomic analyses

In-depth non-targeted proteomic analysis identified a total of 8741 proteins at 1% FDR, and 7664 of which (87.7%) consisted of ≥3 peptides (Supplementary Fig. 1). Non-targeted proteomic analysis from the same PBMC was performed in duplicate in seven samples, resulting in the moderate reproducibility of this assay (median correlation coefficient [r] = 0.80 [range, 0.78–0.84]) (Supplementary Fig. 2A, B).

QuantSeq 3’ mRNA sequencing (RNA-seq) analysis was performed in 74 samples, including the discovery cohort of 60 patients with IBMFS and 14 healthy controls, and full-length RNA-seq analysis was performed on 18 samples, comprising 10, 4, and four patients with monoallelic *SBDS* variants, biallelic *SBDS* variants, and ADH5/ALDH2 deficiency, respectively. The number of proteins identified in the non-targeted proteomic analysis that overlapped with the QuantSeq 3’ RNA-seq and full-length RNA-seq analyses was also assessed. Of the total of 7664 identified proteins with ≥3 peptides, 6451 (84.2%) with full-length RNA-seq analysis and 6045 (78.9%) overlapped with mRNA expression measured with QuantSeq 3’ RNA-seq (Supplementary Fig. 3A). Of these, 5929 protein or mRNA expression levels with available expression data for all analyses were used for subsequent comparative analysis.

The correlation between non-targeted proteomic and QuantSeq 3’ RNA-seq expression levels was positive for 72.2% of protein–mRNA pairs in 74 samples, including 60 samples of the discovery cohort and 14 healthy controls, with a mean Spearman’s correlation coefficient of 0.114 (Supplementary Fig. 3B). When evaluating different biological processes, the correlation between protein and mRNA expression levels was highest for specific pathways, such as hematopoietic cell lineage, cell adhesion, and ribosomal (Supplementary Fig. 3C). Furthermore, transcriptome-based deconvolution analysis using bulk mRNA expression data from the discovery cohort was performed in CIBERSORTx [28] to assess the cell-type-specific gene expression profiles in PBMC. No significant cell fractionation imbalance was observed between each disorder (Supplementary Fig. 4).

### Non-biased clustering based on proteomic analysis for IBMFS

**Fig. 3:**
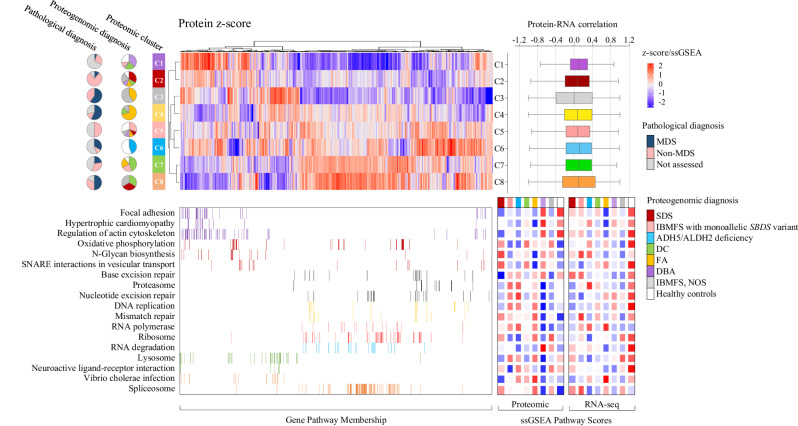
Unsupervised proteomic-based cluster for IBMFS subtypes. Proteomic cluster and differentially expressed proteins. Each row represents a proteomic cluster, and each column represents a protein. Red/blue indicates up/down expression patterns of different proteins within clusters. The pie charts indicate the percentage of the pathological progression to MDS and IBMFS subtypes in each of eight proteomic-based clusters (C1–C8). The gene members of the major pathways enriched in the gene cluster (heatmap below) are shown. For each pathway, the mean sample-specific gene set enrichment analysis (ssGSEA) scores in the respective IBMFS, based on proteomic and transcriptomic data, are shown in the right. Correlations between protein and mRNA expression levels are indicated for each proteomic cluster. ADH5 alcohol dehydrogenase 5, ALDH2 aldehyde dehydrogenase 2, DBA Diamond–Blackfan anemia, DC dyskeratosis congenita, FA Fanconi anemia, GSEA gene set enrichment analysis, IBMFS inherited bone marrow failure syndrome, MDS myelodysplastic syndrome, SDS Shwachman–Diamond syndrome.

Using non-targeted proteomic profiling on 74 samples obtained from 60 patients with IBMFS and 14 healthy controls, unsupervised clustering identified eight independent proteomic clusters (C1–C8), each providing proteogenome-based disease classification and pathological diagnosis (Fig. 3). Patients with DBA, SDS, FA, ADH5/ALDH2 deficiency, and DC were enriched in C1, C2/C8, C3/C4, C6, and C7 clusters, respectively. Then, the distinctive pathways enriched in each of the clusters were evaluated, and a significant downregulation of proteins involved in the KEGG ribosome and spliceosome pathways were particularly characteristic of C1 and C2, enriched in patients with DBA and SDS, respectively; the FDR q-value for ribosome and spliceosome pathways in GSEA was <0.0001 for both clusters. Proteins involved in DNA replication and mismatch repair pathways were enriched in C6 cluster, a characteristic of ADH5/ALDH2 deficiency (FDR *q*-values, 0.039 and 0.039, respectively). Upregulation of the p53 signaling pathway was previously reported in DBA [29]; however, it was not significant in C1 that was enriched by DBA in this cohort. The C3, C4, and C8 clusters included a significantly higher proportion of patients with pathologic MDS than the other clusters (*P* = 5.00 × 10^−4^).

### Diagnosis of IBMFS through proteomic analysis

**Fig. 4:**
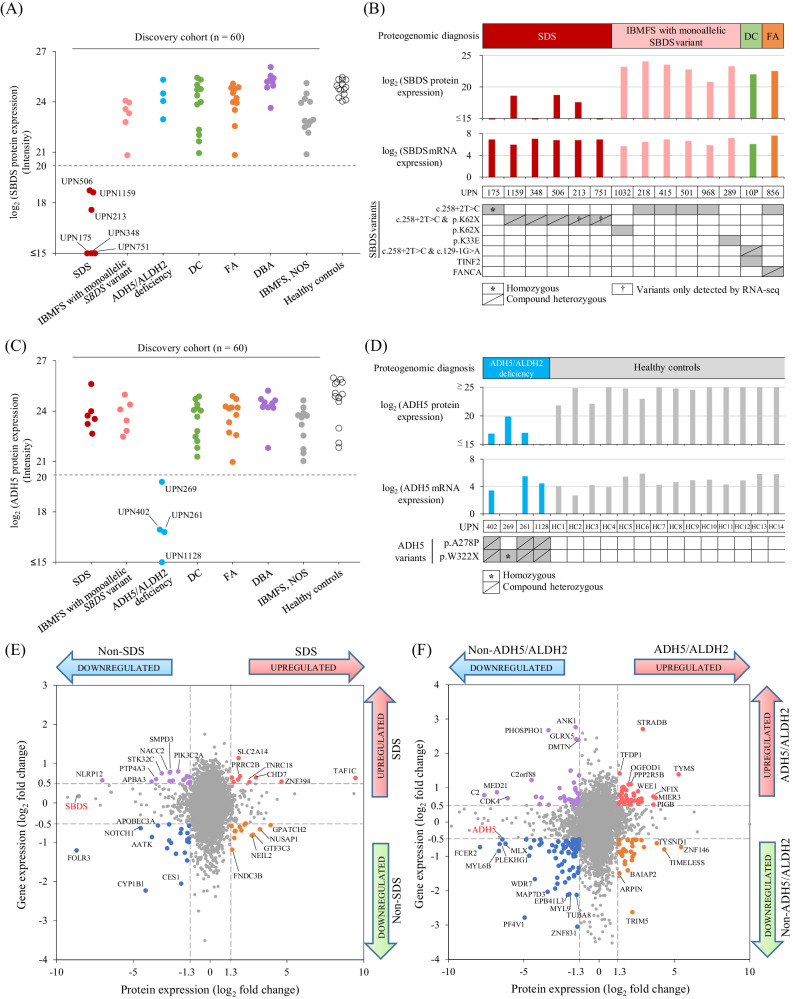
Proteogenomic-based diagnostic testing for SDS and ADH5/ALDH2 deficiency. **A** SBDS protein expression in each IBMFS group. Four patients carrying biallelic *SBDS* pathogenic variants had decreased SBDS protein expression. In patients with IBMFS with monoallelic *SBDS* pathogenic variants (*n* = 10), two (UPN213 and UPN751) had significantly decreased the SBDS protein expression. No patients in other IBMFS groups had decreased SBDS protein expression. **B** Differences in the SBDS protein and *SBDS* mRNA expression based on individual *SBDS* variants. **C** ADH5 protein expression in each IBMFS group. **D** Differences in the ADH5 protein and *ADH5* mRNA expression based on individual *ADH5* variants. Starburst plot integrating proteomic and mRNA expression analyses between patients with and without SDS (**E**), and between those with and without ADH5/ALDH2 deficiency (**F**). ACTB actin beta, ADH5 alcohol dehydrogenase 5, ALDH2 aldehyde dehydrogenase 2, DBA Diamond–Blackfan anemia, DC dyskeratosis congenita, FA Fanconi anemia, IBMFS inherited bone marrow failure syndrome, LCL lymphoblastoid cell line, NOS not otherwise specified, UPN unique patient number, RPM reads per million, SDS Shwachman–Diamond syndrome.

The characteristic markers of protein and mRNA expressions with significant differences were evaluated for each IBMFS disorder (Supplementary Table 6). In the discovery cohort, 6 of 60 patients with IBMFS had significantly decreased SBDS protein expression, whereas 14 healthy controls showed no decrease in its expression (Fig. 4A). Western blotting of lymphoblastoid cell lines (LCLs) derived from patients with SDS and healthy controls confirmed the consistency with proteomic analysis (Supplementary Fig. 5A, B). In six patients with decreased SBDS protein expression, four (UPN175, UPN348, UPN506, and UPN1159) harbored biallelic *SBDS* variants detected in the short-read DNA-seq analysis, and the remaining two (UPN213 and UPN751) harbored monoallelic pathogenic *SBDS* variant detected in DNA-seq (Fig. 4B). For these two patients, full-length RNA-seq alignment reads detected both allele variants, suggesting that they were consistent with the results of the non-targeted proteomic analysis. Among the eight patients with monoallelic *SBDS* variants who did not have protein loss, only variants detected by DNA-seq could be identified in the RNA-seq read sequences (Supplementary Figs. 6A, B).

Starburst plots are developed for the expression of 5929 assessable mRNAs and proteins between the SDS samples (*n* = 6) and non-SDS samples including healthy controls (*n* = 68). Although the *SBDS* mRNA expression was not downregulated in patients with SDS, its protein expression was consistently reduced, indicating the advantage of measuring SBDS proteins to diagnose SDS (Fig. 4E and Supplementary Table 6). Similarly, non-targeted proteomic analysis in 18 samples of BMNC revealed that two patients with SDS (UPN175 and UPN894) with biallelic *SBDS* variants had significantly decreased SBDS protein expressions (*P* = 2.90 × 10^−4^, Supplementary Figs. 7A, B). Subsequently, to assess the *SBDS* mRNA expression in hematopoietic stem cells (HSC) fractions, a single-cell RNA-seq on Lin^-^CD34^+^ HSCs fractions was performed in a patient with SDS (UPN175, *n* = 1) and healthy controls (*n* = 4) (Supplementary Fig. 8A). As with the transcriptomic analysis of PBMC samples, the *SBDS* mRNA expression was not significantly reduced for each HSC fraction (Supplementary Fig. 8B–C).

Next, a diagnostic system for ADH5/ALDH2 deficiency can be established by evaluating a digenic disorder caused by the combination of biallelic *ADH5* variants and *ALDH2* polymorphism (rs671), differentially expressed proteins and mRNAs, between patients with (*n* = 4) and without (*n* = 70) ADH5/ALDH2 deficiency. Four patients with ADH5/ALDH2 deficiency had a significantly reduced ADH5 protein expression (*P* = 9.81 × 10^−3^), whereas the ADH5 protein expression in the remaining 56 patients with IBMFS and 14 healthy controls was normal (Fig. 4C). Western blotting of LCLs from two patients with ADH5/ALDH2 deficiency demonstrated a defective ADH5 protein, consistent with the results of proteomic analysis (Supplementary Fig. 5C, D). No difference in ALDH2 protein expression levels was observed between patients with and without ADH5/ALDH2 deficiency (Supplementary Fig. 9A). Moreover, neither *ADH5* nor *ALDH2* mRNA expression was significantly correlated between patients with and without ADH5/ALDH2 deficiency (Fig. 4D, Supplementary Figs. 5C and 9B). Starburst plots of differentially expressed proteins and mRNA indicated that ADH5 protein expression is one of the most useful markers as compared to other proteins and mRNA expressions and that measuring the ADH5 protein is a more suitable diagnostic assay than *ADH5* mRNA expression (Fig. 4F). Similarly, we performed an integrated analysis of significantly differentially expressed proteins and mRNA expressions between patients with DC (*n* = 12) and non-DC (*n* = 62), FA (*n* = 11) and non-FA (*n* = 63), and DBA (*n* = 9) and non-DBA (*n* = 65) (Supplementary Figs. 10–12 and Supplementary Table 6).

### Establishment of a rapid diagnostic system for SDS and ADH5/ALDH2 deficiency

**Fig. 5:**
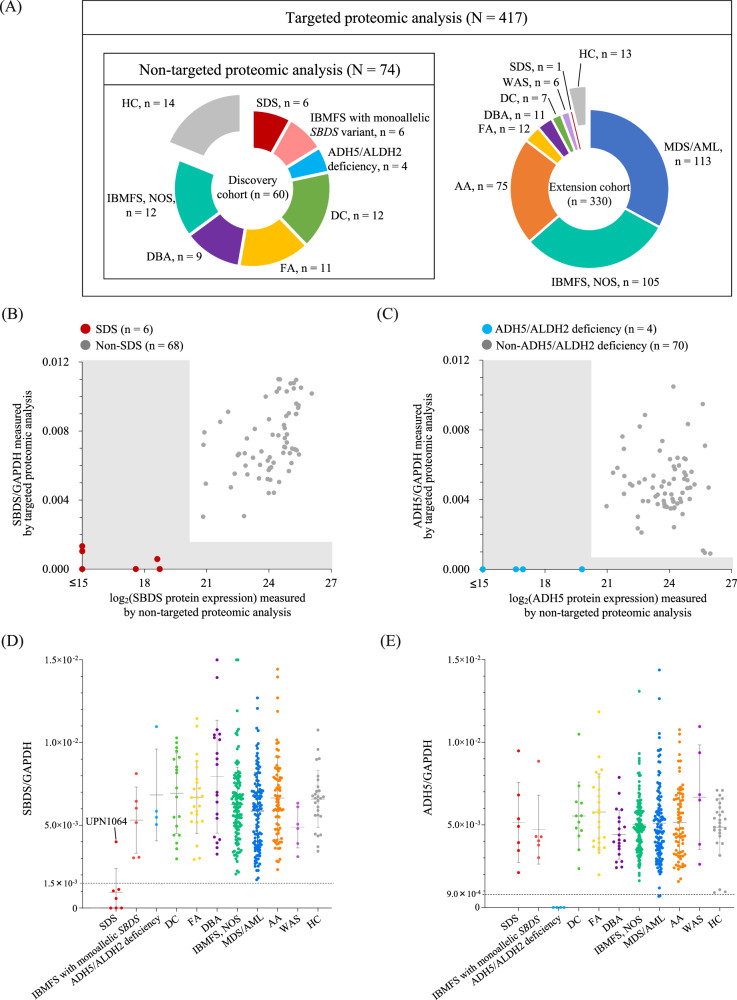
Potential utility of targeted proteomic analysis using IBMFS-related small panels. **A** Summary of the discovery and extension cohort. Non-targeted proteomic analysis was performed on the discovery cohort (*n* = 60) and healthy controls (HC, *n* = 14). Targeted proteomic analysis was performed on 417 samples from the discovery cohort (*n* = 60), the extension cohort (*n* = 330), and HC (*n* = 27). Comparison of SBDS (**B**) and ADH5 (**C**) protein expression levels measured by non-targeted and targeted proteomic analyses. **D**, **E** SBDS and ADH5 protein expression levels measured by targeted proteomic analysis in 417 samples from patients with IBMFS-related hematological disorders (*n* = 390) and HC (*n* = 27). In both measurement assays, SBDS (**D**) and ADH5 (**E**) protein expressions were significantly decreased in patients with SDS and ADH5/ALDH2 deficiency, respectively (*P* < 0.001 each). AA aplastic anemia, ADH5 alcohol dehydrogenase 5, ALDH2 aldehyde dehydrogenase 2, BMNC bone marrow mononuclear cell, DBA Diamond–Blackfan anemia, DC dyskeratosis congenita, FA Fanconi anemia, HC Healthy controls, IBMFS inherited bone marrow failure syndrome, MDS/AML myelodysplastic syndrome/acute myeloid leukemia, NOS not otherwise specified, PBMC peripheral blood mononuclear cells, SDS Shwachman–Diamond syndrome, UPN unique patient number, WAS Wiskott–Aldrich syndrome.

To provide a large-scale rapid screening system for IBMFS in a practical clinical setting, targeted proteomic analysis was performed using a small panel to detect SBDS, ADH5, WASP, and GAPDH proteins. Figure 5A and Supplementary Table 2 show the characteristics of 417 patients from the discovery and expansion cohorts, which include those with IBMFS-related hematologic diseases and healthy controls. In each confirmed patient sample, the SBDS and ADH5 protein expression levels were consistently very low in both targeted and non-targeted proteomic analyses (Fig. 5B, C).

In a total 417 samples, SBDS protein expression levels were significantly low in patients with SDS (*P* = 4.98 × 10^−9^) (Fig. 5D and Supplementary Fig. 13A, B). Similarly, ADH5 and WASP protein expression levels were significantly reduced in patients with ADH5/ALDH2 deficiency and WASP, respectively (*P* = 1.66 × 10^−6^ and *P* = 4.36 × 10^−7^) (Fig. 5E, Supplementary Fig. 13C–F, and Supplementary Fig. 14). Furthermore, the cutoff SBDS and ADH5 protein expression levels were determined as the mean minus two standard deviations (1.5 × 10^−3^ and 9.0 × 10^−4^ each) in the 417 samples excluding SDS and ADH5/ALDH2 deficiency, respectively; the sensitivity and specificity were 85.7% and 93.4% in SBDS and 100.0% and 97.5% in ADH5, indicating the sufficient diagnostic utility as a large-scale screening tool to identify patients requiring early identification and therapeutic intervention.

## Discussion

The first multi-omics analysis integrating a comprehensive proteogenomic and transcriptomic profiling was conducted for various patients with IBMFS. The comprehensive proteomic-based clustering was generally consistent with the molecular diagnosis and revealed specific pathway abnormalities of each IBMFS group. The SBDS and ADH5 protein expression levels were significantly decreased in patients with SDS and ADH5/ALDH2 deficiency, respectively, indicating the diagnostic utility of proteomic analysis for these patients. In a total of 417 samples with IBMFS or associated hematological disorders, targeted proteomic analysis was found to provide a simple and direct diagnostic contribution for both patients with SDS and ADH5/ALDH2 deficiency. Clinically accessible PBMC and BMNC samples have different percentages of each cell, depending on the clinical status of each patient; however, the use of these samples would be highly beneficial for patients with suspected IBMFS, as they would provide a simpler and quicker screening test.

Conventional short-read NGS analysis sometimes overlooks patients with SDS due to the misalignment of NGS reads derived from the homologous *SBDSP1* pseudogene [11]. Since the monoallelic *SBDS* variant with wildtype allele insufficiently causes SDS, whether these variants occur on *cis* or *trans* alleles in patients with two *SBDS* variants should be manually evaluated [19]. Western blotting and/or long-read NGS analysis is generally necessary to provide an accurate diagnosis [11, 13, 30]. These procedures were labor-intensive and time-consuming; thus, developing these assays as a rapid and simple large-scale screening tool seems impractical. To the best of our knowledge, no simple rapid screening assay has been developed for diagnosing SDS to date, and thus, this proteomic-based diagnostic system would be feasible to rapidly administered large-volume sample screening with sufficient sensitivity and specificity for the early identification and therapeutic intervention.

The majority of IBMFS, including SDS and ADH5/ALDH2 deficiency, exhibit a predisposition to developing myeloid malignancy or solid tumors. The definitive IBMFS diagnoses could affect the initiation of cancer screening and directly on urgent treatment decisions and cancer prevention. Approximately 4% of young-adult patients with MDS aged 18–40 years carried *SBDS* variants, and the majority of them had never been diagnosed with SDS until MDS occurred, indicating a potentially large number of patients who are overlooked during childhood [31–33]. Clinical outcomes of patients with SDS and myeloid malignancies are exceptionally poor due to high therapy-related toxicities and relapse incidence even with hematopoietic stem cell transplantation (HSCT) [13, 34]. The preceding registry data indicated improved survival outcomes for patients with SDS who undergo routine bone marrow surveillance and receive HSCT before developing overt myeloid malignancies [31, 35]. Early identification of SDS using proteomic-based screening assay would help establish the appropriate therapeutic intervention and improved the disease prognosis.

In UPN1064 of the extension cohort, DNA-seq identified compound heterozygous *SBDS* splice site and missense variants (c.258+2T>C and c.97A>G, p.K33E). However, the targeted proteomic analysis showed a normal SBDS protein expression (Fig. 5D). This patient presented with a normal karyotype (46,XX) but with skeletal anomaly and fatty stools, diagnosed with SDS. Peptide fragments derived from the loss-of-function SBDS protein caused by this missense variant (p.K33E) were presumably detected by proteomic analysis. Similarly, two patients with WAS (UPN241D and IBMFS-Pro-755) had pathogenic hemizygous *WAS* splice site and missense variants (c.360+1G>A and c.223G>A, p.V75M), respectively. However, the WAS protein expression did not decrease (Supplementary Fig. 14). Some aberrant proteins from the missense or splice site variants can be challenging to distinguish from wildtype proteins in the proteomic assays. Moreover, specific techniques for identifying peptide fragments derived from single nucleotide substitutions will be developed in the future. In addition, *ALDH2* rs671 is a common SNP in the Asian populations, and evaluating not only the ADH5 protein expression but also the *ALDH2* genotyping simultaneously would be required to diagnose ADH5/ALDH2 deficiency.

This study has several limitations. First, due to the small number of patients of each IBMFS in the discovery cohort, diagnostic tests based on proteomic analysis of IBMFS other than SDS and ADH5/ALDH2 deficiency could not be established. However, for example, patients with DC had increased TINF2, ACD, and POT1 protein expression levels, which are components of the shelterin complex and involved in the telomere protection (Fig. 2), which may lead to the development of new diagnostic tests as the number of patients increases. Second, the heterogeneity of stored specimens from multicenter sites used in this study may have influenced the lack of high correlation between the protein and gene expression levels. Future large-scale studies using large numbers of fresh clinical specimens are needed to evaluate the possibility of developing a rapid, reproducible, and comprehensive clinical diagnostic test for IBMFS based on proteomic analysis.

Conclusively, the first proteogenomic analysis was performed in patients with IBMFS and identified eight independent proteomic clusters associated with IBMFS subtypes and characteristic pathways. Furthermore, the clinical application of targeted proteomic assays constructed from these results may help diagnose and screen IBMFS, including SDS and ADH5/ALDH2 deficiency, for which appropriate clinical screening tests are lacking.

### Supplementary information

Supplemental Information

Supplemental Tables

## Data Availability

All data needed to evaluate the conclusions are present in the paper and/or the supplemental information. The raw sequences are not publicly available due to privacy concerns. However, they are available from the corresponding authors (H.M, hideki-muramatsu@med.nagoya-u.ac.jp) upon reasonable request and with permission of the Institutional Review Board of the Nagoya University Graduate School of Medicine involved.
