# Supplementary material for: Integrated proteogenomic analysis for inherited bone marrow failure syndrome
Source: Leukemia. 2024 May 13;38(6):1256–65. doi: 10.1038/s41375-024-02263-1 (PMC11147772; doi:10.1038/s41375-024-02263-1)
Supplement: Supplementary file 1 — Supplemental Information [file 41375_2024_2263_MOESM1_ESM.docx]

**SUPPLEMENTARY INFORMATION**

**Supplemental Notes　　　　　　 pp. 2–5**

Case presentations of patients with biallelic or monoallelic *SBDS* variants in the discovery cohort

**Supplemental Methods** **pp. 5–6**

**Supplemental References** **pp. 6–7**

**Supplemental Figures pp. 8–21**

Figure S1. Identified peptide counts detected by non-targeted proteomic analysis.

Figure S2. Evaluating the reproducibility of non-target proteome analysis.

Figure S3. Integration of proteomic and transcriptomic analyses in the discovery cohort.

Figure S4. Cell-type deconvolution using bulk RNA sequencing in the discovery cohort.

Figure S5. Western blotting of SBDS and ADH5 proteins.

Figure S6. Alignment sequence reads of the full-length RNA sequencing and target-captured DNA sequencing analysis.

Figure S7. SBDS protein expression of BMNC in IBMFS.

Figure S8. *SBDS* mRNA expression in HSC fraction using single-cell RNA sequencing.

Figure S9. Comparison between ALDH2 protein and *ALDH2* mRNA expressions in the discovery cohort.

Figure S10. Integration of proteomic and transcriptomic analyses between DC and non-DC.

Figure S11. Integration of proteomic and transcriptomic analyses between FA and non-FA.

Figure S12. Integration of proteomic and transcriptomic analyses between DBA and non-DBA.

Figure S13. ROC curve based on targeted proteomic analysis.

Figure S14. WAS protein expression measured by targeted proteomic analysis.

**Supplemental Tables (uploaded as an independent file)**

Table S1. Patient characteristics of the non-targeted proteomic analysis using BMNC.

Table S2. Clinical characteristics of patients with targeted proteomic analysis.

Table S3. Peptide information in targeted proteomic analysis.

Table S4. List of detected variants in the discovery cohort.

Table S5. Clinical presentation of patients with biallelic or monoallelic pathogenic *SBDS*

variants.

Table S6. Summary of significant proteins and mRNAs for each IBMFS group.

**Case presentations of patients with biallelic or monoallelic *SBDS* variants in the discovery cohort**

**Patients with biallelic *SBDS* variants**

*UPN175*

UPN175 exhibited neutropenia, short stature (−2.0 standard deviation [SD]), and severe diarrhea and was referred to our hospital for a detailed evaluation at 5 years of age. Her laboratory data were as follows: white blood cell (WBC) count, 1.4 × 10^9^/L; hemoglobin (Hb), 10.2 g/dL; and platelet counts (Plt), 120.0 × 10^9^/L. The neutrophil count was markedly decreased to 0.4 × 10^9^/L. A homozygous splice site variant in the *SBDS* gene (c.258+2T>C) was detected in target-captured next-generation sequencing (NGS) analysis. An extremely low SBDS protein expression was observed in non-targeted proteomic analysis. An abnormal karyotype of 46,XY,del(20)(q11.2q13.3) was found in the G-banding test. She did not require transfusion dependency; however, at 29 years of age, she progressed to myelodysplastic syndromes (MDS) and is currently preparing for hematopoietic stem cell transplantation (HSCT).

*UPN348*

UPN348 presented recurrent upper respiratory tract infections at 5 months of age. She presented mild anemia (Hb 7.8 g/dL) and neutropenia (1.4 × 10^9^/L), but not accompanied with growth retardation. Hypoplastic marrow with prominent red blood cell dysplasia was observed in the bone marrow smear; hence, she was diagnosed with MDS. Target-captured NGS analysis identified biallelic *SBDS* variants (c.258+2T>C and p.K62X) with abnormal SBDS protein expression; thus, she was finally diagnosed with SDS. The patient has progressed to MDS in children and is carefully monitored with bone marrow examination while preparing for HSCT.

*UPN506*

UPN506 had a WBC of 2.6 × 10^9^/L, Hb of 10.3 g/dL, low Plt of 27.0 × 10^9^/L, and extremely low neutrophil count of 0.3 × 10^9^/L. Physical findings were characterized by a short stature. At 7 of age years, he tested positive for monosomy 7 and trisomy 8 in the bone marrow examination and fluorescence in situ hybridization (FISH), respectively, suggesting the progression to MDS without an obvious increase of the blast cells. Biallelic *SBDS* variants (c.258+2T>C and p.K62X) were observed in target-captured NGS analysis, and thereby, the patient was finally diagnosed with SDS. He has not developed transfusion dependency; however, he is carefully followed up and is preparing for HSCT.

*UPN1159*

UPN1159 presented a short stature with a SD of −2.9, chronic liver disorder, autism spectrum disorder, abnormal pancreatic exocrine function, diarrhea symptoms, and thrombocytopenia at the age of 3 years. The telomere length was 1.71 SD, within the normal range. Biallelic *SBDS* variants (c.258+2T>C and p.K62X) were detected in target-captured NGS analysis, and a decreased SBDS protein expression was observed in proteomic analysis. The alignment read of RNA-seq identified biallelic variants of the *SBDS* gene (c.258+2T>C and p.K62X); hence, the patient was finally diagnosed with SDS and was carefully followed up with bone marrow examinations.

*UPN751*

UPN751 presented thrombocytopenia and neutropenia at the age of 18 months. Her laboratory data were as follows: WBC, 4.6 × 10^9^/L; Hb, 5.7g/dL; Plt, 9.0 × 10^9^/L; and a slightly low neutrophil count, 0.1 × 10^9^/L. Persistent diarrhea and hepatosplenomegaly were also reported, and her bone marrow examination revealed a pathological diagnosis of MDS. A monoallelic *SBDS* variant (c.258+2T>C) was identified in target-captured NGS analysis; however, her SBDS protein expression was confirmed to be defective by non-targeted proteomic analysis. The RNA-seq alignment read identified biallelic variants of the *SBDS* gene (c.258+2T>C and p.K62X), leading to the final diagnosis of SDS. She showed transfusion dependency and has been alive after HSCT from an HLA-matched unrelated bone marrow donor with conditioning regimens of melphalan, fludarabine, and total body irradiation of 4 Gy.

*UPN213*

At birth, UPN213 had no obvious external malformations but presented several complications: short stature (−2.0 SD), severe neutropenia, fatty stools, and low fat-soluble vitamins. His laboratory data were as follows: normal WBC, 8.1 × 10^9^/L; Hb, 9.2 g/dL; Plt, 32.0 × 10^9^/L; and slightly low neutrophil counts, 0.3 × 10^9^/L. Although he had no transfusion dependency, only 3% of blast cells were detected in the bone marrow at the age of 1 year. A monoallelic *SBDS* variant (c.258+2T>C) was identified in target-captured NGS analysis, whereas a decreased SBDS protein expression was observed in non-targeted proteomic analysis. Moreover, the alignment read derived from RNA-seq demonstrated biallelic *SBDS* variants (c.258+2T>C and c.183_184delTAinsCT, p.K62X). He was finally diagnosed with SDS. His brother was also diagnosed with SDS but developed acute myeloid leukemia and died after HSCT due to relapse.

**IBMFS with monoallelic pathogenic *SBDS* variants**

*UPN1032*

UPN1032 had abnormal T-cell receptor excision circle values indicating a severe condition on combined immunodeficiency newborn screening test at birth. Physical manifestations included growth and developmental delay, which was later confirmed as trisomy 21 by the G-banding test. Target-captured NGS analysis and RNA-seq alignment reads identified a monoallelic pathogenic *SBDS* variant (p.K62X), and non-targeted proteomic analysis showed a normal SBDS protein expression. She had no persistent neutropenia or additional physical symptoms associated with SDS, was finally diagnosed with non-SDS, and was undergoing continuous follow-up.

*UPN218*

A 17-month-old girl presented with neutropenia and diarrhea. No physical malformations or growth retardation was observed. She presented WBC of 10.9 × 10^9^/L, Hb of 12.2 g/dL, Plt of 17.5 × 10^9^/L, and completely absent neutrophils; however, she had a hyperplastic cellular bone marrow without MDS findings. Moreover, a normal karyotype (46,XX) was detected in the G-banded chromosome analysis. Target-captured NGS analysis and RNA-seq alignment reads identified a monoallelic pathogenic *SBDS* variant (c.258+2T>C), and non-targeted proteomic analysis confirmed a normal SBDS protein expression; thus, she was finally diagnosed with non-SDS.

*UPN289*

UPN289 was born at 36 weeks of gestation, weighing 1,672 g with intrauterine growth restriction. At 10 months of age, she developed epilepsy and was treated with valproic acid (VPA), an antiepileptic drug. At 12 months of age, she presented a gradually decreased Plt count, and bone marrow examination showed a hypoplastic marrow, no megakaryocytes, and a moderate dysplasia in all lineages. Her pancytopenia persisted even after changing her antiepileptic drug from VPA to levetiracetam. Subsequently, target-captured NGS analysis and RNA-seq alignment reads identified a monoallelic pathogenic *SBDS* variant (c.97A>G, p.K33E), and non-targeted proteomic analysis revealed a normal SBDS protein expression; thus, the patient was finally diagnosed with non-SDS.

*UPN415*

UPN 415 presented with purpura on the lower extremities at 6 months of age. He had a slightly decreased WBC of 1.9 × 10^9^/L, Hb 8.0 g/dL, Plt 13.0 × 10^9^/L, and a slightly low neutrophil count of 0.1 × 10^9^/L at 46 months of age. The telomere length in peripheral blood lymphocytes was within the normal range at −0.93 SD. Bone marrow examination revealed an extremely hypoplastic marrow without megakaryocytes and pseudo-Pelger–Huët anomaly was identified in the peripheral blood (PB) and the bone marrow (BM) smear. Target-captured NGS analysis and RNA-seq alignment reads found a monoallelic pathogenic *SBDS* variant (c.258+2T>C), and non-targeted proteomic analysis revealed a normal SBDS protein expression; thus, the patient was finally diagnosed with non-SDS.

*UPN501*

At birth, UPN501 showed a low Plt count of 21.0 × 10^9^/L and bloody stools. Her thrombocytopenia persisted, and thereby, she remained transfusion dependent. Mild hypoplastic marrow was detected in the BM smear. Only a small number of megakaryocytes had isolated nuclei without myeloid or erythroid lineage dysplasia; thus, the patient was clinically diagnosed with congenital thrombocytopenia. A monoallelic *SBDS* variant (c.258+2T>C) was identified in target-captured NGS analysis and RNA-seq alignment reads, whereas normal SBDS protein expression was observed in non-target proteomic analysis.

*UPN968*

At 13 months of age, UPN968 presented with a short stature, skeletal abnormalities, and neutropenia. Her laboratory data showed a WBC of 8.4 × 10^9^/L, Hb of 11.3 g/dL, Plt count of 42.0 × 10^9^/L, and neutrophil count of 0.4 × 10^9^/L. The BM smear detected normoplasia without dysplasia or increased blast cell counts. Target-captured NGS analysis and RNA-seq alignment reads found a monoallelic pathogenic *SBDS* variant (c.258+2T>C), and non-targeted proteomic analysis revealed a normal SBDS protein expression. Further, western blotting was performed in proteins from patient-derived lymphoblastoid cell lines to assess the SBDS protein expression, and we also confirmed its normal protein expression. Finally, the patient was diagnosed with non-SDS.

*UPN10P*

At the age of 2 years, UPN10P presented bilateral exudative retinopathy, severe thrombocytopenia, and mental retardation, although the telomere length of PB lymphocytes was not shortened (+0.87 SD). Target-captured NGS analysis identified a pathogenic *TINF2* variant (c.C844T, p.R282C) and two splice site variants of *SBDS* (c.129-1G>A and c.258+2T>C). The splice site variant of the *SBDS* gene in c.129-1G>A is a very rare variant and is not registered in the SNP database of gnomAD and without pathogenic reports[^1^](#_ENREF_1). Therefore, whether these two splice site variants were present in the same allele using long PCR sequencing was evaluated, and monoallelic mutations were confirmed. The non-targeted proteomic analysis also revealed a normal SBDS protein expression. Based on these molecular analyses and clinical phenotypes, this patient was finally diagnosed with Revesz syndrome, a rare type of dyskeratosis congenita.

*UPN856*

At 18 months of age, UPN856 presented moderate thrombocytopenia and was followed up for immune thrombocytopenia. Her WBC was 7.9 × 10^9^/L; Hb, 13.1 g/dL; Plt, 261.0 × 10^9^/L; and neutrophil count, 2.6 × 10^9^/L. At 4.5 years of age, Plt counts gradually decreased to 50.0–90.0 × 10^9^/L; however, no therapeutic intervention was performed. Target-captured NGS analysis and RNA-seq alignment reads identified a monoallelic pathogenic *SBDS* variant (c.258+2T>C) and a monoallelic pathogenic *FANCA* variant (c.2546delC, p.S849FfsX40). This patient showed a missense gene variant (c.T2470C, p.C824R) in *FANCA* not registered as a pathogenic gene variant by DNA-seq and not registered in the SNP database[^1^](#_ENREF_1) . Non-targeted proteomic analysis showed normal SBDS protein expression, and detailed examinations revealed a positive chromosomal fragility test, which led to a final diagnosis of Fanconi anemia.

**Supplemental Methods**

*Target-captured DNA-seq analysis*

Genomic analysis of PB/BM samples was performed using target-captured DNA-seq covering 184 IBMFS-related genes or the whole exome. Sequence library construction and bioinformatics analysis of variants and copy number alterations were performed as previously described[^2^](#_ENREF_2). A variant allele frequency of >0.3 (30%) was used as the cut-off value. The American College of Medical Genetics criteria[^3^](#_ENREF_3) defined causal variants as pathogenic or likely pathogenic.

*QuantSeq 3’ mRNA* *and full-length RNA sequencing analysis*

QuantSeq 3’ RNA-seq was performed using all PBMC samples from the discovery cohort (n = 60), and full-length RNA-seq analysis was conducted in 18 patients from the discovery cohort, including 14 patients carrying monoallelic or biallelic *SBDS* variants. According to the manufacturers’ instructions, QuantSeq 3’ RNA-seq and full-length RNA-seq libraries were prepared using a QuantSeq-pool sample-barcoded 3’ mRNA library prep kit (Lexogen, Vienna, Austria) and NEBNext Ultra II Directional RNA Library Prep Kit (New England Biolabs, Inc., Ipswich, Massachusetts, USA), respectively. Expression profiles obtained using QuantSeq 3’ RNA-seq were calculated following the recommendation of the data analysis, including the removal step of redundant reads generated during PCR using unique molecular identifiers (https://github.com/Lexogen-Tools/). Raw count data of the full-length RNA-seq for each gene were normalized by variance-stabilizing transformation using DESeq2[^4^](#_ENREF_4). To estimate the relative fraction of each cell type in the discovery cohort, the cell-type-specific gene expression profiles were analyzed using CIBERSORTx^[5](#_ENREF_5)^.

*Immunoblotting*

Lymphoblastoid cell lines (LCLs) were generated using PBMC samples from patients with SDS (UPN175 and UPN751), monoallelic pathogenic *SBDS* variant (UPN968), ADH5/ALDH2 deficiency (UPN261 and UPN402), and healthy controls. Western blotting of SBDS and ADH5 proteins was performed in triplicate using the JESS technology (ProteinSimple, San Jose, CA, USA) with a 12–230 kDa separation module (ProteinSimple). Primary antibodies were used at the following dilutions: mouse anti-SBDS (1:50 dilution, sc-271350, Santa Cruz Biotechnology, Dallas, Texas, USA), rabbit anti-ADH5 (1:50 dilution, Invitrogen, Waltham, Massachusetts, USA), and mouse anti-beta actin (1:250 dilution, sc-47778, Santa Cruz Biotechnology). Anti-mouse and anti-rabbit dedicated secondary antibodies (ProteinSimple) were used without dilution.

*Single-cell RNA sequencing*

Single-cell RNA-seq analysis was performed in five BMNC samples with SDS (UPN 894, n = 1) and healthy controls (n = 4). Lineage-negative and CD34-positive fractions from BMNC were sorted using FACSAria™ Fusion (BioLegend, San Diego, California, USA) with anti-human CD34 antibody (BioLegend) and anti-human Lineage Cocktail (BioLegend). Using the Chromium Next GEM Single-Cell 3’ Reagent kit v3.1 (10x Genomics, Pleasanton, California, USA), cDNA libraries were generated based on the manufacturer’s instructions. The alignment, barcode processing, and UMI counting of the sequencing data were performed using 10× cell ranger (version 6.1.2) (10x Genomics). The downstream work was performed using the R package Seurat (version 4.0.6)[^6^](#_ENREF_6).

*Telomere length measurement*

Telomere length shortening was assessed by measuring the telomere length using PB lymphocytes of patients suspected of IBMFS and AA as described elsewhere[^7^](#_ENREF_7). The telomere length was measured with flow-FISH using a Telomere PNA Kit (Dako Cytomation, Glostrup, Denmark).

*Morphological evaluation*

PB/BM specimens obtained from patients in the discovery cohort were examined by adopting the European Working Group of MDS criteria for the morphological evaluation of pediatric MDS[^8^](#_ENREF_8). These were evaluated by physicians (AH, MI, and HI) with expertise in pediatric hematological morphology.

*Statistical analysis*

To compare the frequency of variants or other clinical features between disease groups, the χ^2^ test was used to analyze categorical variables, and Mann–Whitney U-test was used to analyze continuous variables. Correlation coefficients were calculated using Spearman’s rank correlation. EZR (Saitama Medical Center, Jichi Medical University), a graphical user interface designed for R statistical software (The R Foundation for Statistical Computing, Vienna, Austria), was used to perform statistical analyses[^9^](#_ENREF_9).

**Supplemental References**

1. Chen S, Francioli LC, Goodrich JK, Collins RL, Kanai M, Wang Q*, et al.* A genome-wide mutational constraint map quantified from variation in 76,156 human genomes. *bioRxiv* 2022**:** 2022.2003.2020.485034.

2. Muramatsu H, Okuno Y, Yoshida K, Shiraishi Y, Doisaki S, Narita A*, et al.* Clinical utility of next-generation sequencing for inherited bone marrow failure syndromes. *Genetics in medicine : official journal of the American College of Medical Genetics* 2017 Jul; **19**(7)**:** 796-802.

3. Richards S, Aziz N, Bale S, Bick D, Das S, Gastier-Foster J*, et al.* Standards and guidelines for the interpretation of sequence variants: a joint consensus recommendation of the American College of Medical Genetics and Genomics and the Association for Molecular Pathology. *Genetics in medicine : official journal of the American College of Medical Genetics* 2015 May; **17**(5)**:** 405-424.

4. Love MI, Huber W, Anders S. Moderated estimation of fold change and dispersion for RNA-seq data with DESeq2. *Genome biology* 2014; **15**(12)**:** 550.

5. Newman AM, Steen CB, Liu CL, Gentles AJ, Chaudhuri AA, Scherer F*, et al.* Determining cell type abundance and expression from bulk tissues with digital cytometry. *Nature biotechnology* 2019 Jul; **37**(7)**:** 773-782.

6. Hao Y, Hao S, Andersen-Nissen E, Mauck WM, 3rd, Zheng S, Butler A*, et al.* Integrated analysis of multimodal single-cell data. *Cell* 2021 Jun 24; **184**(13)**:** 3573-3587 e3529.

7. Miwata S, Narita A, Okuno Y, Suzuki K, Hamada M, Yoshida T*, et al.* Clinical diagnostic value of telomere length measurement in inherited bone marrow failure syndromes. *Haematologica* 2021 Sep 1; **106**(9)**:** 2511-2515.

8. Cantu Rajnoldi A, Fenu S, Kerndrup G, van Wering ER, Niemeyer CM, Baumann I*, et al.* Evaluation of dysplastic features in myelodysplastic syndromes: experience from the morphology group of the European Working Group of MDS in Childhood (EWOG-MDS). *Ann Hematol* 2005 Jul; **84**(7)**:** 429-433.

9. Kanda Y. Investigation of the freely available easy-to-use software 'EZR' for medical statistics. *Bone marrow transplantation* 2013 Mar; **48**(3)**:** 452-458.

**Figure S1. Identified peptide counts detected by non-targeted proteomic analysis.**

0

20

40

60

80

100≤

0

200

600

1,000

1,400

1,200

Number of proteins (n)

Identified peptide counts (n)

3

7,664 proteins (≥3 identified peptides) counts)

8,741 proteins

400

800

Identified peptide counts detected in non-targeted proteomic analysis. Non-targeted proteomic analysis identified 8,741 proteins at a 1% protein false discovery rate. Of the 8,741 proteins, 7,664 (87.7%) protein values consisted of ≥3 peptides.

**Figure S2. Evaluating the reproducibility of non-target proteome analysis.**

(**A**) Correlation plots of proteomic expression levels in duplicates. The same seven PBMC samples were used to perform non-targeted proteomic analysis in duplicate (r = Pearson’s correlation coefficient). (**B**) Nonbiased clustering analysis using proteomic profiling in two different batches (batches A and B). PBMC, peripheral blood mononuclear cells; UPN, unique patient number.

**Figure S3. Integration of proteomic and transcriptomic analyses in the discovery cohort.**

(**A**) Numbers of proteins overlapping the Quant 3’ mRNA and full-length RNA sequencing analyses among 7,664 proteins that were detected by non-targeted proteomic analysis for ≥3 peptides. (**B**) Spearman’s rank order correlation between the protein and the mRNA abundance. The correlation between the protein and mRNA expression levels was assessed in 74 samples, including 60 samples of the discovery cohort and 14 healthy controls. The correlation was positive for 72.2% protein–mRNA pairs in the discovery cohort with a mean Spearman’s correlation coefficient of 0.11. (**C**) The correlation between the protein and mRNA expression levels was highest for specialized pathways, such as the hematopoietic cell lineage, cell adhesion pathway, and ribosomal pathway.

**Figure S4. Cell-type deconvolution using bulk RNA sequencing in the discovery cohort.**

(**A**) Cell-type deconvolution using bulk RNA sequencing in the discovery cohort. Gene expression profiles were imputed, and cell-type abundances were estimated using CIBERSORTx. (**B**–**J**) The proportion of various cell types was assessed among IBMFS groups of the discovery cohort. ADH5, alcohol dehydrogenase 5; ALDH2, aldehyde dehydrogenase 2; DBA, Diamond–Blackfan anemia; DC, dyskeratosis congenita; FA, Fanconi anemia; IBMFS, inherited bone marrow failure syndrome; NOS, not otherwise specified; SDS, Shwachman–Diamond syndrome.

**Figure S5. Western blotting of SBDS and ADH5 proteins.**

(**A**) Correlation of *SBDS* mRNA and SBDS protein expressions. (**B**) Western blotting of the SBDS protein expression. Using LCLs (UPN175, UPN751, UPN968, and healthy control), the SBDS expression was abnormal in UPN175 and UPN751, whereas the SBDS protein expression was normal in UPN968 and healthy control, consistent with the results of proteomic analysis. (**C**) Correlation of *ADH5* mRNA and ADH5 protein expressions. (**D**) Western blotting of the ADH5 protein expression using LCLs (UPN261, UPN402, UPN856, and healthy control). The ADH5 expression was absent in UPN261 and UPN402, whereas the ADH5 protein expression was normal in UPN856 and healthy control. ACTB was used as the internal control. ACTB, actin beta; ADH5, alcohol dehydrogenase 5; ALDH2, aldehyde dehydrogenase 2; FA, Fanconi anemia; LCLs, lymphoblastoid cell lines; SDS, Shwachman–Diamond syndrome; UPN, unique patient number.

**Figure S6. Alignment sequence reads of the full-length RNA sequencing and target-captured DNA sequencing analysis.**

(**A–B**) In two (UPN213 and UPN751) of the six patients with SDS, target-captured DNA analysis could not identify biallelic *SBDS* variants because the reads with c.183_184TA>CT were disregarded as low-quality reads in both patients. Alignment of full-length RNA sequencing indicated that both patients harbored a compound heterozygote for c.258+2T>C and c.183_184TA>CT alleles. Chr, chromosome; NGS, next-generation sequencing; SDS, Shwachman–Diamond syndrome; UPN, unique patient number.

**Figure S7. SBDS protein expression of BMNC in IBMFS.**

(**A**) SBDS protein expression levels using BMNC samples of patients with IBMFS (n = 13) and healthy controls (n = 5). (**B**) The SBDS protein expression was significantly decreased in patients with SDS (UPN175 and UPN894, n = 2) than those without SDS (n = 16). BMNC, bone marrow mononuclear cells; DBA, Diamond–Blackfan anemia; HC, healthy control; IBMFS, inherited bone marrow failure; NOS, not otherwise specified; SDS, Shwachman–Diamond syndrome; UPN, unique patient number.

**Figure S8. *SBDS* mRNA expression in HSC fraction using single-cell RNA sequencing.**

(**A**) Single-cell RNA sequencing (scRNA-seq) based on cell-type annotation of patients with SDS (UPN894). Lineage-negative CD34-positive fractions were isolated from bone marrow mononuclear cells of UPN894, and the *SBDS* gene expression was assessed using scRNA-seq. (**B**–**C**) Moderate *SBDS* gene expression levels were observed in each cell fraction between UPN894 and healthy controls. CMP, common myeloid progenitor; GMP, granulocyte/macrophage progenitors; HSC, hematopoietic stem cell; MEP, megakaryocyte/erythrocyte progenitor; SDS, Shwachman–Diamond syndrome; UPN, unique patient number.

**Figure S9. Comparison between ALDH2 protein and *ALDH2* mRNA expressions in the discovery cohort.**

(**A**) ALDH2 protein expression in each IBMFS group. (**B**) Comparison between ALDH2 protein and *ALDH2* mRNA expressions in the discovery cohort. Blue circles showed each patient with ADH5/ALDH2 deficiency. This *ALDH2* variant (p.E504K, rs671) was not affected by *ALDH2* mRNA and its protein expression levels, respectively. ADH5, alcohol dehydrogenase 5; ALDH2, aldehyde dehydrogenase 2; DBA, Diamond–Blackfan anemia; DC, dyskeratosis congenita; FA, Fanconi anemia; IBMFS, inherited bone marrow failure syndrome; NOS, not otherwise specified; RPM, reads per million; SDS, Shwachman–Diamond syndrome.

**Figure S10. Integration of proteomic and transcriptomic analyses between DC and non-DC.**

Differentially expressed proteins and genes between DC (n = 12) and non-DC (n = 62) were evaluated. Starburst plots indicated differentially expressed mRNAs and proteins between patients with and without DC based on 3' QuantSeq and proteomic analysis. Pink circles indicate the significantly upregulated proteins and mRNAs in DC, whereas dark blue circles show the significantly downregulated proteins and mRNAs in DC. DC, dyskeratosis congenita.

**Figure S11. Integration of proteomic and transcriptomic analyses between FA and non-FA.**

Differentially expressed proteins and genes were assessed between FA (n = 11) and non-FA (n = 63). Starburst plots indicated differentially expressed mRNAs and proteins between patients with and without FA based on 3' QuantSeq and proteomic analysis. Pink circles indicate the significantly upregulated proteins and mRNAs in FA, whereas dark blue circles show significantly downregulated proteins and mRNAs in FA. FA, Fanconi anemia.

**Figure S12. Integration of proteomic and transcriptomic analyses between DBA and non-DBA.**

Differentially expressed proteins and genes were assessed between DBA (n = 9) and non-DBA (n = 65). Starburst plots indicated differentially expressed mRNAs and proteins between patients with and without DBA based on 3' QuantSeq and proteomic analysis. Pink circles indicate significantly upregulated proteins and mRNAs in DBA, whereas dark blue circles show significantly downregulated proteins and mRNAs in DBA. DBA, Diamond–Blackfan anemia.

**Figure S13. ROC curve based on the targeted proteomic analysis.**

Comparison of each protein expression. SBDS (**A**), ADH5 (**C**), and WASP (**E**) protein expressions were assessed. The upper and lower bars indicate the maximum and minimum values in each protein expression, respectively, and the middle bar is the average. ROC curves were also evaluated in patients with SDS (**B**), ADH5/ALDH2 deficiency (**D**), and WAS (**F**). SDS, Shwachman–Diamond syndrome; ROC, receiver operating characteristic; WAS, Wiskott–Aldrich syndrome.

**Figure S14. WAS protein expression measured by targeted proteomic analysis.**

(**A**) WAS protein expressions measured by targeted proteomic analysis. Patients with WAS had a significantly reduced WAS protein expression, except for two patients (IBMFS-Pro-755 and UPN241D) with normal WAS protein expression. (**B**) The *WAS* variant landscape. IBMFS-Pro-755 harboring a hemizygous *WAS* variant (c.223G>A, p.V75M), and UPN241D with a *WAS* splice site variant (c.360+1G>A) showed normal WAS protein expressions. AA, aplastic anemia; DBA, Diamond–Blackfan anemia; FA, Fanconi anemia; IBMFS, inherited bone marrow failure syndromes; MDS, myelodysplastic syndromes; NOS, not otherwise specified; SDS, Shwachman–Diamond syndrome; UPN, unique patient number; WAS, Wiskott–Aldrich syndrome.
